# IgG1 as a Potential Biomarker of Post-chemotherapeutic Relapse in Visceral Leishmaniasis, and Adaptation to a Rapid Diagnostic Test

**DOI:** 10.1371/journal.pntd.0003273

**Published:** 2014-10-23

**Authors:** Tapan Bhattacharyya, Armon Ayandeh, Andrew K. Falconar, Shyam Sundar, Sayda El-Safi, Marissa A. Gripenberg, Duncan E. Bowes, Caroline Thunissen, Om Prakash Singh, Rajiv Kumar, Osman Ahmed, Osama Eisa, Alfarazdeg Saad, Sara Silva Pereira, Marleen Boelaert, Pascal Mertens, Michael A. Miles

**Affiliations:** 1 Faculty of Infectious and Tropical Diseases, London School of Hygiene and Tropical Medicine, London, United Kingdom; 2 Departamento de Medicina, Universidad del Norte, Barranquilla, Colombia; 3 Institute of Medical Sciences, Banaras Hindu University, Varanasi, Uttar Pradesh, India; 4 Faculty of Medicine, University of Khartoum, Khartoum, Sudan; 5 Coris BioConcept, Gembloux, Belgium; 6 Immunology and Infection Laboratory, Queensland Institute of Medical Research, Herston, Queensland, Australia; 7 Department of Laboratory Medicine, Karolinska Insitutet, Stockholm, Sweden; 8 Department of Public Health, Institute of Tropical Medicine, Antwerp, Belgium; Barcelona Centre for International Health Research (CRESIB), Spain

## Abstract

**Background:**

Visceral leishmaniasis (VL), caused by protozoa of the *Leishmania donovani* complex, is a widespread parasitic disease of great public health importance; without effective chemotherapy symptomatic VL is usually fatal. Distinction of asymptomatic carriage from progressive disease and the prediction of relapse following treatment are hampered by the lack of prognostic biomarkers for use at point of care.

**Methodology/Principal Findings:**

All IgG subclass and IgG isotype antibody levels were determined using unpaired serum samples from Indian and Sudanese patients with differing clinical status of VL, which included pre-treatment active VL, post-treatment cured, post-treatment relapsed, and post kala-azar dermal leishmaniasis (PKDL), as well as seropositive (DAT and/or rK39) endemic healthy controls (EHCs) and seronegative EHCs. *L. donovani* antigen-specific IgG1 levels were significantly elevated in relapsed *versus* cured VL patients (p<0.0001). Using paired Indian VL sera, consistent with the known IgG1 half-life, IgG1 levels had not decreased significantly at day 30 after the start of treatment (p = 0.8304), but were dramatically decreased by 6 months compared to day 0 (p = 0.0032) or day 15 (p<0.0001) after start of treatment. Similarly, Sudanese sera taken soon after treatment did not show a significant change in the IgG1 levels (p = 0.3939). Two prototype lateral flow immunochromatographic rapid diagnostic tests (RDTs) were developed to detect IgG1 levels following VL treatment: more than 80% of the relapsed VL patients were IgG1 positive; at least 80% of the cured VL patients were IgG1 negative (p<0.0001).

**Conclusions/Significance:**

Six months after treatment of active VL, elevated levels of specific IgG1 were associated with treatment failure and relapse, whereas no IgG1 or low levels were detected in cured VL patients. A lateral flow RDT was successfully developed to detect anti-*Leishmania* IgG1 as a potential biomarker of post-chemotherapeutic relapse.

## Author Summary

Visceral leishmaniasis (VL) is a systemic disease with highest prevalence in South Asia, East Africa, and Brazil. VL is caused by protozoan (unicellular) parasites of the *Leishmania donovani* complex, transmitted to humans when an infected sandfly takes a bloodmeal. Within the human host, the parasites replicate within cells, particularly of bone marrow and spleen. Without effective treatment, symptomatic VL is usually fatal. As outlined in a recent World Health Organisation report, the development of new diagnostic tools to test for successful cure after chemotherapy is a research priority. In this work we investigated the association of clinical status of VL patients (active pre-treatment, and those deemed cured or relapsed post-treatment) with subclasses of the IgG antibody response made to *L. donovani* infection. We show that high levels of subclass IgG1 are found in pre-treatment and relapsed patients, but are very much lower in patients deemed to be cured. We further show that the decrease in IgG1 is detectable in patients 6 months after successful treatment, and that this detection method can be adapted to a rapid diagnostic test format requiring minimal technical expertise. Thus we believe that IgG1 levels are potentially a biomarker of post-chemotherapeutic monitoring.

## Introduction

The leishmaniases are widespread neglected infectious diseases of major public health importance, caused by protozoan parasites of the *Leishmania* (*Leishmania*) and *Leishmania* (*Viannia*) subgenera. There are two principal symptomatic clinical presentations of leishmaniasis: a) visceral (kala-azar, VL) caused by the *Leishmania donovani* complex, with circa 400,000 cases per year [1], which without appropriate chemotherapy is usually fatal, and b) cutaneous (CL) caused by diverse *Leishmania* species, some of which may give rise to diffuse cutaneous leishmaniasis (DCL) or metastatic mucocutaneous disease (MCL), the latter with devastating destruction of the nasopharynx [2]. The effective clinical management, chemotherapy and control of transmission of VL are largely dependent on early and unequivocal diagnosis. Given that many VL patients live below the poverty threshold in remote areas poorly serviced by the health system, the diagnostic tools should be ASSURED (Affordable, Sensitive, Specific, User Friendly, Rapid, Equipment free and Deliverable where needed) [3]. The most sensitive and specific method to detect the causative agent of VL is microscopic examination of (invasive) spleen aspirates; bone marrow and lymph node aspirates provide similar high specificity but lesser sensitivity. More user-friendly point-of-care (POC) diagnostics have been developed based on antibody detection against rK39 and these provide high diagnostic accuracy in suspected first-time episodes of VL when combined with a clinical case definition [4]–[6]. However, these rapid diagnostic tests based on antibody detection are unable by themselves to distinguish asymptomatic carriers from those who will progress to acute fatal disease, and following chemotherapy they remain positive for many months precluding the detection of any relapse. To resolve the limitations of current diagnostic tools higher resolution, non-invasive, rapid and affordable POC tests are thus needed that define clinical status and indicate prognosis.

Current serological tests for VL include the enzyme linked immunosorbent assay (ELISA) with either crude or purified *Leishmania* promastigote antigens, the direct agglutination test (DAT) or indirect immunofluorescence test (IFAT) [7], [8]. Each of these tests has disadvantages: the ELISA requires laboratory facilities and technical training; the DAT test has limited commercial access and involves several hours or overnight incubation before reading the results, and the IFAT uses a costly fluorescence microscope.

The recombinant repetitive antigen rK39, a product of the *Leishmania* kinesin-like gene, which was first cloned from Brazilian *Leishmania infantum* (synonym *L. chagasi*) [9] has been adapted to an immunochromatographic, lateral flow format and applied extensively as a rapid diagnostic test (RDT) for VL. However, in multicentre trials rK39 had much higher sensitivity in South Asia than in East Africa [10], [11]. This could be due to the geographical diversity in the 6.5 repeats within the rK39 antigen sequence [12] and/or regional differences in anti-*Leishmania* IgG titres between the different human populations [13]. A modified recombinant derivative of rK39, designated rK28, which incorporated the first two rK39 repeats of a Sudanese kinesin flanked by HASPB1 and HAPB2 repeats, improved serological sensitivity for East African VL patients [14]. The ELISA, DAT, IFAT, rK39 and rK28 serological tests all rely on the detection of anti-*L. donovani* complex IgG. In human VL the IgG is produced secondarily, after IgM. IgG is divided into IgG1, IgG2, IgG3, and IgG4 subclasses, according to their ranked relative abundance in normal serum [15]. In general, human IgG1 and IgG3 are generated in response to protein antigens, whilst IgG2 and IgG4 are instead predominantly produced in response to polysaccharide antigens [15]. Previous studies on the humoral responses during active VL and after treatment, and on post kala-azar dermal leishmaniasis (PKDL), have been performed using samples from multiple endemic regions and a variety of serological tests [16]–[31]. Several of these studies, almost all of which used ELISAs, assessed anti-*L. donovani* complex titres of IgG, its subclasses and other Ig isotypes. In terms of IgG subclasses, increased IgG1 titres were identified in active disease (VL or PKDL) compared to healthy controls, and reduced IgG1 titres were reported after successful VL treatment. This published work suggested that the dynamics between proportionate IgG subclass responses and the clinical status of patients warranted further investigation to search for prognostic VL biomarkers.

A recent WHO report [8] designated the development of new diagnostics to determine cure as a research priority for VL, and there is an additional need for biomarkers to distinguish asymptomatic non-progressors from early progressive VL. Here we explore the potential of differential IgG subclass profiles to provide a biomarker for patients who relapsed after chemotherapy and therefore require urgent follow-up and life-saving additional or alternative chemotherapy. We show that the dynamics and levels of specific IgG1 responses can be indicative of relapse, and can be assessed using a simple, lateral flow immunochromatographic RDT format.

## Methods

### Ethics statement

In India, research on comparative serology and the collection of all serum or plasma samples was approved by the Ethics Committee of the Banaras Hindu University, Varanasi. Similarly, in Sudan research and collection of serum samples was approved by the Ethical Research Committee, Faculty of Medicine, University of Khartoum and the National Health Research Ethics Committee, Federal Ministry of Health. Written informed consent was obtained from all adult subjects included in the study or from the parents or guardians of individuals less than 18 years of age. This research was also approved, as part of the NIDIAG project, by the London School of Hygiene and Tropical Medicine Ethics Committee.

### Sources of sera/plasma

Indian plasma samples were collected after 2007 from active VL, cured, relapsed, PKDL and asymptomatic groups from the endemic region of Muzaffarpur, Bihar state, north-eastern India, and control subjects from a region where VL is not endemic. In India active cases of VL were diagnosed parasitologically by microscopy of splenic aspirates. Sudanese serum samples were collected in 2011 and 2013, from active VL, treated, relapsed, PKDL, and endemic controls in the Gedaref region in eastern Sudan. All patients were HIV negative. Active cases of VL were diagnosed by a combination of microscopy of bone marrow or lymph node aspirates in conjunction with serological assays. We have previously observed that serum and plasma derived from the same sample show no difference in titre in ELISA against *L. donovani* lysate (unpublished observations).

#### Unpaired samples

Table 1 shows the numbers of samples used in unpaired comparisons of IgG subclass responses and clinical status. For the Indian samples, two sets of comparisons were performed using these unpaired samples. There was an initial pilot study (Trial 1), followed by an expanded investigation (Trial 2), using different samples in each case. For the Sudanese sera, only one study was performed, after the Indian pilot serology.

**Table 1:** Single (unpaired) samples used in IgG subclass comparisons, and clinical status of the Indian and Sudanese patient groups.

	VL patient status					Controls		
Unpaired group	Active	Cured	Relapsed	Treated^a^	PKDL	Other diseases	EHC (seropositive)	EHC (seronegative)
Trial 1 India	20	21	19	-	-	20	-	20
Trial 2 (Expanded) India	46	28	35	-	24	28	28	32
Trial Sudan	47	-	-	22	23	-	30	12

EHC = endemic healthy control; PKDL = post kala-azar dermal leishmaniasis.

a treated, not in recent past, but time of treatment unknown.

#### Paired samples and multiple sequential samples

The comparative dynamics of IgG subclass response were studied using paired serum samples or, in the case of PKDL patients, several sequential samples from individual patients. Sera had been collected prospectively and the intervals between collections are shown in Table 2. For the Indian patients samples were available prior to treatment of active VL (day 0), paired with days 15, 30, or approximately 180 after start of treatment, depending on the patient. For Indian PKDL multiple sequential serum samples from the same patient were available at days 0, 30, 60, and 180 or 360 (Table 2). Paired sera from Sudanese patients were taken prior to treatment (day 0) and at the end of treatment lasting 11 days (ambisome), 17 days (sodium stibogluconate (SSG)+paromomycin) or 30 days (SSG alone).

**Table 2:** Paired and multiple sequential sera from Indian and Sudanese VL and PKDL.

	Country and disease status (VL or PKDL)							
Group and sampling	India VL			India PKDL				Sudan VL
Group	1	2	3	4	5	6	7	Sudan
Serum (days)	0 and 30	0 and 180	15 and 180	0–30	0–60	0–180	0–360	0 and 11, 17 or 30
Pairs/sequentials (seq)	24 pairs	32 pairs	43 pairs	2 seq	9 seq	9 seq	1 seq	17 pairs

Day 0 = before start of treatment.

### Antigen preparation

*L. donovani* strain MHOM/IN/80/DD8 isolated from India, and MHOM/SD/97/LEM3458 isolated from Sudan, were cultured in αMEM (Sigma, UK) supplemented as described [32]. Mid-to-late log phase promastigote cultures were washed three times in phosphate-buffered saline (PBS), followed by three cycles of flash-freezing in liquid nitrogen and thawing in a 26°C water bath. Subsequently, these cells were subjected to three 30 seconds, 12-micron, sonication cycles on ice at 30 second intervals using a Soniprep 150 sonicator (MSE, UK). Sonicates were centrifuged at 12000× *g* for 1 minute at 4°C, and the supernatants were used as antigen. Protein concentrations in these lysates were determined using the BCA Protein Assay kit (Fisher Scientific, Loughborough, UK).

### ELISA

Lysates of *L. donovani* DD8 (for Indian samples) or LEM3458 (for Sudanese samples), diluted to 2 µg/ml in 35 mM NaHCO_3_/15 mM NaCO_3_ buffer (pH 9.6), were added at 100 µl/well to Immulon 4HBX ELISA plates (VWR, Lutterworth, UK) and incubated overnight at 4°C. After washing the plates three times using PBS containing 0.05% (vol/vol) Tween 20 (Sigma, Gillingham, UK) (PBST), they were blocked using 200 µl/well PBST containing 2% skimmed milk powder (Premier International Foods, Spalding, UK) (PBSTM) at 37°C for 2 hr. Following three PBST washes, the human sera were added at 1∶400 dilution for the Indian sera or 1∶100 dilution for the Sudanese sera, in PBSTM, and the plates were incubated at 37°C for 1 hr. The different sample concentrations were used due to the significantly lower anti-*Leishmania* IgG response generated by the Sudanese VL patients, as recently described [13]. Following six PBST washes, 100 µl of horseradish peroxidase-conjugated subclass-specific mouse monoclonal antibodies specific for human IgG1 (ab99774), IgG2 (ab99784), IgG3 (ab99829) or IgG4 (ab99817) (Abcam, UK), diluted 1∶5000 for the Indian sera or 1∶1000 for the Sudanese sera, in PBSTM, were added and plates were incubated at 37°C for 1 hr.

Human IgG isotype responses were determined using 100 µl/well of a 1∶5000 dilution for the Indian sera or 1∶2500 dilution for the Sudanese sera, of horseradish peroxidase–conjugated donkey anti-human IgG secondary antibody (709-035-149: Jackson ImmunoResearch, West Grove, USA) with incubation at 37°C for 1 hr.

Following six PBST washes, 50 mM phosphate/citrate buffer (pH 5.0) containing 2 mM *o*-phenylenediamine HCl and 0.007% (vol/vol) H_2_O_2_ (Sigma, UK) was added at 100 µl/well and incubated in the dark at room temperature for 15 minutes. The substrate reactions were then stopped by the addition of 2M H_2_SO_4_ (50 µl/well) and the ELISA plates were read at 490 nm (Spectra Max 190, Molecular Devices, Sunnyvale, USA, or MRX II, Dynex Technologies, Chantilly, USA). These assays were performed on duplicate plates, simultaneously.

### Statistical analyses

ELISAs cut-offs were determined by the mean values plus three standard deviations obtained from the seronegative endemic healthy control serum samples. Statistical analysis on ELISA data (2-tailed unpaired t-test for unpaired samples, and paired t-test for paired/sequential samples, with 95% confidence interval in both cases) was performed using GraphPad Prism version 4.02 for Windows (GraphPad Software, San Diego, California, USA).

### Prototype *L. donovani* antigen-specific IgG1 immunochromatographic RDTs

The prototype lateral flow immunochromatographic tests consisted of a cassette with a nitrocellulose membrane, a sample pad, a conjugate pad and an absorbent pad, backed with a plastic strip. The nitrocellulose membranes were sensitized with crude *L. donovani* (strain LEM3458) antigen lysate produced as described above and anti-human IgG1-specific antibody labelled with colloidal gold was dried on the conjugate pad. This strip was housed in a plastic cassette with two windows: the application well and the test/reading window. Two prototypes of this assay were developed and tested. For prototype 1, 2 µl of serum and 2 µl of buffer were added to the nitrocellulose strip and then 90 µl of buffer was added to the application well. In prototype 2, the application of 2 µl of serum on the strip was followed by the addition of 75 µl of buffer in the application well. The human IgG that migrated over the nitrocellulose membrane reacted with the immobilized target antigens. The anti-human IgG1-specific conjugated MAbs rehydrated by the buffer recognised the antigen-bound human IgG1 in the sample, thereby resulting in a red-purple coloured band. A control line ensured that the human IgG and conjugated anti-human IgG1 migration had occurred successfully. The prototype tests were used with sets of post-chemotherapeutic sera from Indian patients considered to have relapsed VL (n = 30 for prototype 1, of which 22 were also used with prototype 2) or cured VL (n = 21 for prototype 1, of which 5 were also used with prototype 2). Fisher's exact 2-tailed test was used for testing the difference between proportions.

## Results

**Figure 1:**
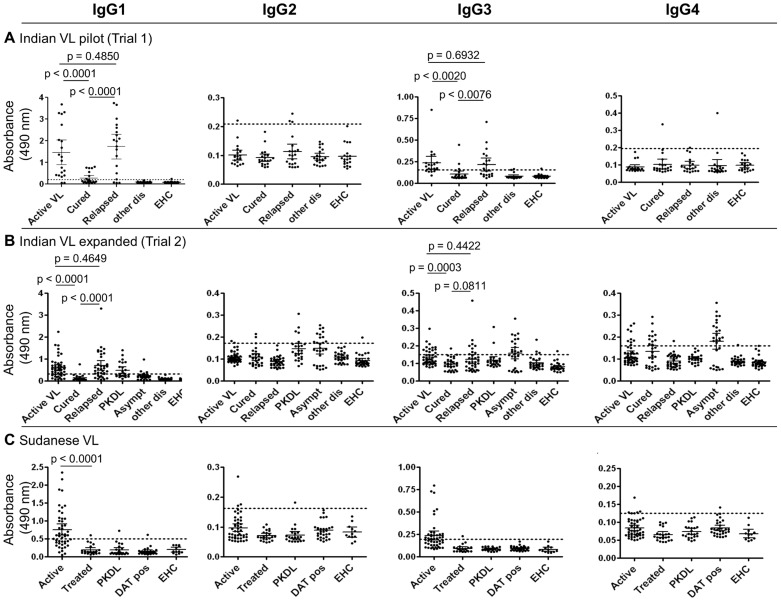
Specific IgG1 ELISA levels were high in active and relapsed VL but negative or substantially decreased in cured VL using unpaired serum samples. [A] Indian VL pilot study (Trial 1). [B] Indian VL expanded study (Trial 2). [C] Sudanese VL. Mean and 95% CI are shown for each data set (solid black lines); note the different Y axis scales. In each study set, the means plus three standard deviations obtained using DAT-seronegative endemic healthy control (EHC) samples was used to calculate the cut-off value (dotted line) and p values of<0.05 were considered significant.

**Figure 2:**
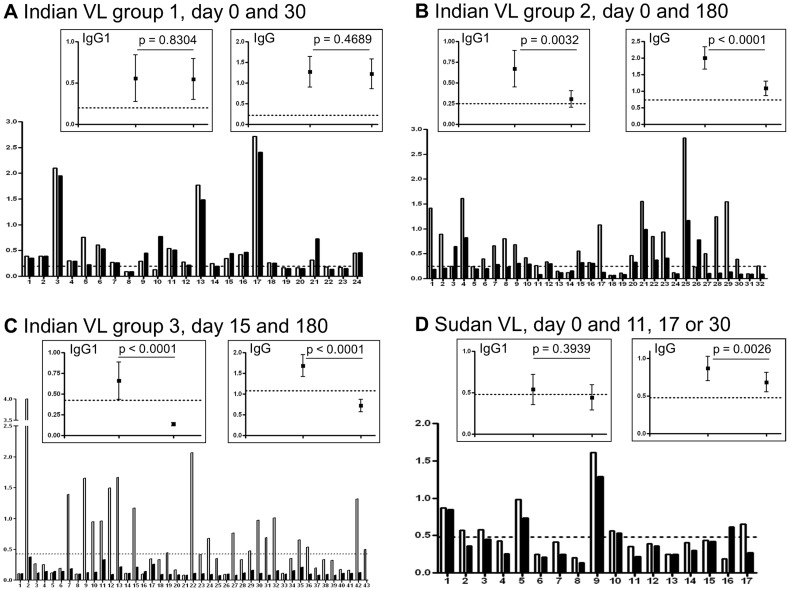
IgG1 decrease following cured VL became more significant with time. [A] Indian paired samples group 1, day 0 and 30. [B] Indian paired samples group 2, day 0 and 180. [C] Indian paired samples group 3, day 15 and 180. [D] Sudanese paired samples, day 0 and after treatment lasting 11, 17 or 30 days. Empty and filled columns represent the earlier and later samples of each pair respectively. Comparison of column heights allows the change in IgG1 level to be seen for each individual patient pre- and post–chemotherapy. For each data set represented in [A]–[D], paired samples from individual patients are presented in the main graph, and in the insets the mean and 95% CI compiled from all patients in that data set are shown for both IgG1 and IgG isotype. Mean plus three standard deviations of the results obtained using the seronegative endemic healthy control samples was used to calculate each cut-off value (dotted line) and p values of<0.05 were considered significant (subclasses IgG2–4 were also assayed but were not informative).

The IgG1 subclass responses gave discrete separations between cured versus active VL and relapsed VL (Figure 1A and 1B). This potential human IgG1 subclass association with clinical VL status was therefore investigated further, in particular to determine whether it could be suitable for development as a rapid (lateral flow) diagnostic test (RDT) to detect relapsed VL patients at point of care (POC). IgG isotype responses showed a similar trend to IgG1; however as illustrated in Figure 2 (see below, paired samples) and already noted in the literature [33]–[37] this was less apparent and was inconsistent.

### In Indian VL IgG1 levels were high pre-treatment and in relapse but were negative or substantially diminished in cured VL patients

Figure 1A and Table S1 show the results of the initial pilot study of IgG subclass in unpaired Indian VL patients with different clinical status. There was marked elevation of IgG1 levels in active (untreated) VL patients; IgG1 levels were dramatically reduced in patients who were treated and considered to be cured by clinical and parasitological assessments. Thus, 90.0% (18/20) pre-treatment active VL patients were serologically positive, above the cut-off value, whereas only 33.3% (7/22) patients considered to be cured remained positive, all of whom had low IgG1 titres (p<0.0001, cured VL *versus* active or relapsed VL; Figure 1A). However, the IgG1 levels in the patients who were unsuccessfully treated were high and at levels comparable to the group of patients with active VL prior to treatment (p = 0.485; Figure 1A; Table S1). This trend for IgG1 levels was partially mirrored by IgG3. The IgG3 levels were however lower in the active VL patients (IgG1: 90.0% positive: IgG3: 75.0% positive) and the relapsed VL patients (IgG1: 84.2% positive: IgG3: 52.6% positive) patients and 47.4% (9/19) of these relapsed VL patients were IgG3 negative (Figure 1A; Table S1). In comparison IgG2 profiles were very weak (active VL: 5.0% positive; relapsed VL: 15.8% positive; cured VL: 0.0% positive) and IgG4 levels were negative or at the cut off boundary, except for one cured patient and one in the other disease group (a meningitis patient) (Figure 1A).

### An expanded study of Indian VL confirmed that elevated IgG1 levels were a feature of relapse

Based on the initial study showing that elevated IgG levels were a potential biomarker of Indian VL treatment failure and relapse, a larger number of unpaired samples from Indian VL patients were analysed, together with those from a post kala-azar dermal leishmaniasis (PKDL) group, and from a group of patients who were VL asymptomatic but all of whom had positive DAT and/or rK39 serology. Since this second larger cohort was investigated after the initial study (Trial 1), the results are presented separately in Figure 1B, but were also incorporated into Table S1. In Trial 2 similar results were obtained to those in Trial 1, with 67.4%, 71.4% and 3.6% of the active VL, relapsed VL and cured VL patients, respectively, being IgG1 positive (Figure 1B, Table S1). A lower percentage of the cured VL patients were IgG1 positive in Trial 2 compared to Trial 1 (Trial 1: 33.3% positive; Trial 2: 3.6% positive), although the absolute readings for Trial 1 cured were low and adjacent to cut-off borderline. IgG1 detection showed a much greater sensitivity and specificity than IgG3. Thus amongst the four IgG subclasses IgG1 was indicated for potentially identifying VL Indian patients with relapse versus cure after chemotherapy.

### Sudanese VL patients had reduced IgG1 levels after treatment

Comparisons of Sudanese VL IgG subclass responses were performed with unpaired sera from active VL, treated VL and PKDL patients, as well as DAT-positive and DAT–negative endemic healthy controls (Table S1), at 1∶100 dilution to allow for the overall lower IgG titres of Sudanese VL sera compared to Indian sera [13]. A slightly lower percentage of the Sudanese active VL patients (57.4%) had positive specific IgG1 levels compared to the Indian patients (Trial 1: 90.0%; Trial 2: 67.4%), but the treated Sudanese VL patients had low positive IgG1 levels (4.6%) similar to the cured VL patients in the Indian Trial 2 (3.6%). Thus the IgG1 levels in Sudanese active VL and Sudanese treated VL were significantly different (p<0.0001; Figure 1C, Table S1).

### Anti-*L. donovani* IgG1 levels were raised in Indian PKDL

IgG1 levels were elevated in Indian PKDL compared to cured patients. Positivity rate was higher in Indian PKDL (Trial 2, 45.8%) than in Sudanese PKDL (4.3%), (Figures 1B and 1C, Table S1), although overall antibody levels in Sudanese VL were also lower, as we have reported previously [13].

### In Indian VL, anti-*L. donovani* specific IgG1 levels decreased in paired samples approximately 6 months after successful treatment

Having determined that specific IgG1 levels were elevated in single serum samples from cases of VL treatment failure but were decreased in cured VL patients, we compared, for single individuals, paired serum samples obtained prior to or at start of treatment and again at subsequent particular times. As shown in Table 2, for Indian VL paired samples from three Indian VL groups were analysed: group 1: day 0 and 30, group 2: day 0 and 180 and group 3: day 15 and 180. *L. donovani* antigen-specific IgG1 levels were not significantly reduced at 30 days after start of treatment (group 1: p = 0.8304; Figure 2A) but were significantly decreased at approximately 180 days after the start of therapy (group 2: p = 0.0032; group 3: p<0.0001; Figures 2B and 2C, respectively). In contrast, in Indian PKDL patients, a significant decrease in specific IgG1 levels was not observed using the sequential (day 0 to 30, 60, 180 or 360) samples (Table 2: groups 4–7).

### In Sudanese VL specific IgG1 levels did not fall immediately after the start of treatment

Assay of IgG1 levels with paired samples from treated Sudanese VL patients (day 0 and 11, 17 or 30) showed that IgG1 subclass responses do not fall precipitously immediately after start of treatment (Figure 2D), as with Indian VL patients, which also showed no rapid fall in IgG1 level shortly after start of treatment (Figure 2A). However, with one exception, in which titre increased (patient 16, Figure 2D) all Sudanese VL patients showed a non-significant trend for titre to decrease slightly.

### Prototype immunochromatographic rapid diagnostic tests could detect specific IgG1 levels indicative of relapsed VL

**Figure 3:**
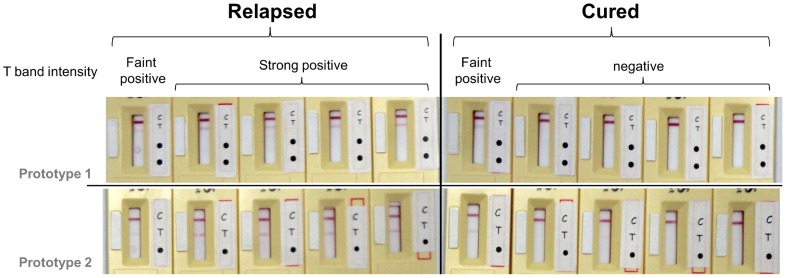
RDT IgG1 prototypes show the ability to distinguish relapsed VL from cured VL. C = migration control line; T = test line. Black dots indicate places where samples should be deposited: 2 µl serum in front of upper dot for prototype 1 or single dot in prototype 2, and 2 µl of buffer in front of lower dot in prototype 1.

Two prototype immunochromatographic (lateral flow) RDTs were designed (see Methods) and tested to determine whether they could be used to discriminate between relapsed and cured VL patients by the detection of *L. donovani* antigen-specific IgG1 levels. The results for some of these patients are shown in Figure 3. As summarised in Table 3, most of the serum samples provided clear positive IgG1 results for the relapsed VL patients. Thus with prototype 1, 25 of 30 (83.3%) relapsed VL patients were positive but only 4 of 21 (19.0%) cured VL patients. Similarly, with prototype 2, 19 of 23 (82.6%) relapsed VL patients were positive but only 1 of 5 (20%) cured patients (p<0.0001 for the prototypes 1 and 2 cumulative results, Fisher's exact 2-tailed test). None of these 5 cured patients tested with either prototype 1 or 2 gave strong positive IgG1 signals. One of patients with malaria and no diagnosed VL had detectable IgG1. Thus, these prototype *L. donovani* antigen-specific IgG1 lateral flow assays could clearly discriminate between most of the relapsed and cured VL patients.

**Table 3:** Summary of results from IgG1 rapid diagnostic tests (RDT) prototypes.

			RDT results	
Patient Groups	n	RDT Used	Positive	Negative
Relapsed VL^a^	30	Prototype 1	83.3% (25/30)	16.7% (5/30)
	23^b^	Prototype 2	82.6% (19/23)	17.4% (4/23)^c^
Cured VL	21	Prototype 1	19.0% (4/21)	81.0% (17/21)
	5^d^	Prototype 2	20.0% (1/5)	80.0% (4/5)^c^
Other diseases^e^	7	Prototype 1	14.3% (1/7)	85.7% (6/7)

a Therapy: sodium antimony gluconate n = 8; miltefosine n = 10; amphotericin B n = 3; combination therapy n = 2.

b 22 of these samples were also used with prototype 1.

c these samples were also negative with prototype 1.

d these 5 samples were also used with prototype 1.

e malaria n = 3; hepatitis n = 1; TB n = 2; dengue n = 1.

## Discussion

Diagnosis of VL is not straightforward: clinical symptoms may overlap with other infectious diseases associated with fever syndrome; parasitological methods of diagnosis are invasive and have limited sensitivities. The currently recommended rK39-based rapid diagnostic tests have their limitations as they cannot distinguish systematically and reliably between the different clinical phases of VL. A large Indian/Nepalese population study reported an association between higher DAT and/or rK39 titres and risk of progression from asymptomatic to symptomatic VL [38]. Recent papers have studied asymptomatic (seropositive) populations in Bangladesh [39] and elsewhere [40]–[42]. In Bangladesh when assessing a patient cohort at 24 months follow-up for VL disease development, discrepancies were found between the molecular and serological tests [39]. In one long-term follow-up of 55 rK39 seropositive asymptomatic cases in India 69% developed VL and 31% remained asymptomatic [43]. However, the proportion of *L. donovani* complex seropositive asymptomatic individuals that progresses to symptomatic VL can be minor, and varies between endemic regions, for example between 1: 2.4 in Sudan [44], 4∶1 in Kenya [45], 8∶1 in Brazil [46], 4∶1 in Bangladesh [47], 8.9∶1 in India and Nepal [48], and 50∶1 in Spain [49]. There is no rapid diagnostic test that determines which asymptomatic carriers will progress to active VL. Nor is there a rapid diagnostic test that is a biomarker of treatment failure and relapse as opposed to cure after chemotherapy. Thus development of such point of care tests has been identified by WHO as a research priority [8].

We are by no means the first to investigate the dynamics of antibody response in the different clinical phases and evolution of VL. The persistence of detectable anti-*Leishmania* IgG years after treatment, mainly using DAT and rK39, has been reported from India [33], [34], Brazil [35], and Sudan [36], [37]. We are not the first to mention that IgG subclass profiles may be associated with clinical status. The evaluation of IgG subclasses has been applied in other parasitic infections including echinococcosis [50]–[52], toxoplasmosis [53], [54], and malaria [55]–[57]. Table 4 summarises previous published studies on IgG subclasses and clinical status of VL.

**Table 4:** IgG subclass serology in VL and PKDL: Published studies.

Reference	Origin of samples	Antigen (assay)	Authors' reports
[16]	Sudan	Intact promastigote (ELISA)	Elevated IgG1 and IgG3 in VL, not IgG2 or IgG4 (n = 15).
[17]	Sudan	Crude promastigote sonicate (ELISA)	Overall decrease in IgG1 and IgG3 (n = 28) 1 month post treatment of VL with sodium stibo-gluconate (Pentostam)
[18]	India	Crude promastigote sonicated lysate (ELISA); whole promastigote (immunoblotting)	IgG1>G2>G3>G4 in VL (n = 10) and PKDL (n = 6). IgG3 recognition of antigens by immunoblot persisted 24 weeks after successful chemotherapy, whereas IgG1 decreased in VL 24 weeks post chemotherapy.
[19]	Venezuela	Promastigote soluble extract (ELISA)	IgG1 predominant subclass; IgG4 also detectable (n = 10).
[20]	Somalia	Crude promastigote lysate (ELISA & western blot)	Elevated IgG1, IgG3, IgG4 in VL (n = 22).
[21]	India	Crude promastigote lysate (ELISA)	IgG1>IgG2>IgG3 = IgG4 before sodium antimony gluconate treatment in responders (n = 10) and non-responders (n = 10). 4–6 weeks post treatment, responders decreased all subclasses; non-responders no significant decrease.
[22]	India	Leishmanial membrane antigens (ELISA)	IgG1 predominant subclass, and IgG3 is useful diagnostic marker, in VL (n = 25).
[23]	India	Leishmanial membrane antigens (ELISA)	IgG1 increase in non-responders to sodium stibogluconate, reduced after subsequent cure by amphotericin B therapy (n = 5); all IgG subclasses decrease in sodium stibogluconate responders (n = 10)
[24]	Brazil, Colombia, Venezuela	Recombinant kinetoplastid membrane protein-1 (ELISA)	IgG1>>IgG3>IgG2>IgG4 in pre-treatment in VL (n = 12)
[25]	Ethiopia	Sonicated promastigote antigen (ELISA)	High IgG1 in VL (n = 10) compared to subclinical DAT positive (n = 18) and successfully treated (n = 20). IgG2 non-discriminating.
[26]	India	Leishmanial membrane antigens (western blot)	IgG1 50 days after sodium antimony gluconate therapy VL patients (n = 7) gave similar but less intense western blotting banding patterns.
[27]	India	Leishmanial membrane antigens (ELISA)	IgG1 elevated in PKDL (n = 23). IgG4 elevated in active VL (n = 10) but not in PKDL (n = 23). IgG1, IgG2, IgG3 overall higher in PKDL (n = 23) than in cured VL (n = 10)
[28]	India	Crude promastigote lysate (ELISA)	IgG1 elevated in VL (n = 38) compared to PKDL (n = 27); IgG2, IgG4 higher in PKDL than VL. IgG3 and IgG4 higher in paediatric (n = 16) than adult VL (n = 22). All IgG subclass levels comparable in paediatric (n = 7) and adult PKDL (n = 20)
[29]	India	Crude promastigote lysate (ELISA)	IgG1 and IgG3 decreased in VL 1 month post amphotericin B treatment (n = 6). Less IgG1 and IgG3 in macular (n = 5) than polymorphic PKDL (n = 11)
[30]	Brazil	Fixed *L. infantum* (syn. *L. chagasi*)promastigote (immunofluorescent flow cytometry)	IgG1>IgG3 in untreated VL (n = 21); absence of IgG2 and IgG4. IgG1 100% sensitive and specific for discriminating pre- and 12 month post-amphotericin B treatment paired sera of patients considered cured.
[31]	India	Crude promastigote lysate (ELISA)	IgG3>>IgG1>>IgG4>IgG2 in polymorphic PKDL (n = 3). IgG3>>IgG1>IgG2≥IgG4 in macular PKDL (n = 11) IgG1 and IgG3 decreased post treatment of polymorphic PKDL (n = 15)

Here, using comparative plate ELISAs and in the context of previous literature, we have specifically examined the capacity of anti-*L. donovani* IgG subclass antibodies to act as a biomarker of therapeutic failure and relapse as opposed to cure. Furthermore, and most importantly, we have shown that the biomarker can be adapted to a lateral flow rapid diagnostic test suitable for use at point of care.

We analysed the IgG subclass profiles of patients in India with active VL and treated VL in comparison with asymptomatic seropositives, PKDL cases, other infectious diseases and endemic healthy controls. Future work could include a wider range of other diseases, including fungal infections. In a pilot study, we saw a remarkable decline in IgG1 levels in samples from unpaired Indian patients who were treated six months previously and were considered to be cured, so much so that the IgG1 titres for almost all of the individual patients fell below the ELISA cut off value for seropositivity (Figure 1A). IgG1 is produced in response to protein antigens and its decline with cure is thus presumably due to disappearance of the antigenic stimulus. A similar profile was to some extent also seen with IgG3, although IgG3 was not consistently raised above the cut-off in active VL. IgG2 levels were low across all groups (Figure 1). The results were similar for Sudan, in that there was a significantly lower level of IgG1 in non-recently treated patients, in almost all cases to below ELISA cut-off, compared to pre-treatment patients (p<0.0001; Figure 1C), although overall IgG and subclass titres were much lower for Sudanese active VL than Indian, as reported previously [13]. Other authors have referred to high IgG1 levels in active VL compared to healthy controls, and a decrease in IgG1 following successful therapy, as assessed by ELISA [17], [21], [23], [29] or in one case by flow cytometry [30].

To explore the timing of the decline in IgG1 levels following successful chemotherapy of Indian VL we compared IgG1 ELISA titres prior to treatment, shortly after the start of treatment or approximately 180 days later, using paired Indian samples (groups 1–3, Table 2). At day 30 after start of treatment (group 1, Table 2) decline in IgG1 was minimal and not significant (p = 0.8304; Figure 2A), which is not surprising as the half life of human IgG1 is estimated to be around 21 days [58]. The slow decline in IgG1 shortly after treatment was confirmed with paired sera from treated Sudanese patients who had active VL (p = 0.3939; Table 2, Figure 2D).

We have not performed a western blot analysis with sera taken at different time points after treatment to determine whether the decrease in IgG1 titres relates to response to particular *L. donovani* antigens. However, one published study comparing western blot profiles using subclass specific conjugates and sera taken before and after treatment reported a general decline in band recognition and not the selective disappearance of bands [26]. Here we have used antigen derived from cultured promastigotes. However, in human VL the stage of the *Leishmania* life cycle is the amastigote, and given access to sufficient quantity of amastigote antigens it would be worthwhile to repeat such a comparative western blot study with amastigotes and subclass specific conjugates. In this way it might be possible to identify and subsequently isolate a specific amastigote antigen(s) applicable to determination of cure.

Recently decreases in IgG1 and IgG3 after cure [59] or post-active disease scarring [60] have also been reported for Brazilian cutaneous leishmaniasis (CL), and higher levels of these IgG subclasses in Turkish patients with active CL compared to endemic controls [61].

IgG subclass responses have been reported for experimental murine models of *Leishmania* infection and for canine infections with *L. infantum*, based on FcγR binding [62]. For canine leishmaniasis there are conflicting interpretations of IgG subclass profiles, (reviewed in [63]), reportedly due to confusion in subclass nomenclature of the commercial polyclonals used [63], [64]. A recent study has proposed improvement of comparative studies by categorising canine IgG subclasses against function of their human analogues [65].

The detection of VL relapse following unsuccessful chemotherapy is of special importance because without effective treatment symptomatic VL is considered to be almost invariably fatal. Thus, if relapse is not recognized and followed up with repeated or alternative treatment, patients, who are often relatively isolated in rural endemic regions, will succumb to the disease. We were therefore interested to examine the IgG subclass profiles in unsuccessful treatment and relapse. Notably, IgG1 levels were raised in patients who failed to respond to chemotherapy and were considered to have relapsed 6 months after start of treatment. As in active VL the majority of relapsed patients had IgG1 titres clearly above the ELISA cut-off (Figures 1A and 1B). Such elevation of IgG1 in patients not responding to treatment has been mentioned rarely in the literature [21], [23]. Accordingly, to provide an RDT for point of care application we devised two lateral flow prototypes, with which we assessed IgG1 seropositivity in treatment failure and relapse. Although we had already demonstrated that IgG1 levels were drastically reduced in cure, 6 months after treatment, for comparison we included a set of serum samples from such cured patients. Strikingly, the majority of patients considered to have relapsed were strongly positive in the IgG1 specific RDT, whereas none of the cured patients were strongly positive and the vast majority were entirely negative (Table 3, Figure 3). Further validation is indicated with a one year longitudinal study (since some patients may relapse between 6 months and one year after treatment [66]) and a larger cohort of patients, ideally with baseline application of the RDT prior to treatment, since despite successful treatment, extent of decline of IgG1 might be influenced by the pre-treatment titre. All relapse patients might be screened/rescreened for HIV infection to assess whether relapse may be associated with immunocompromised status. Thus, with optimisation and standardisation of reagents, and preferably with higher discriminative sensitivity, this RDT may provide an important and life-saving epidemiological tool to detect relapse of VL.

We have shown the potential of IgG1 to be a simple indicator of VL clinical status in terms of relapse. Symptomatic VL is treated at an acute phase of infection, which may favour the decline of IgG1 seropositivity in cure; it remains to be seen whether such markers are equally applicable to the treatment of prolonged chronic infections. As PKDL is a long-established chronic infection, this may explain why IgG1 does not decline with time after chemotherapy. A recent study has proposed that a high seropositivity in asymptomatic people may be associated with greater risk of progression to symptomatic VL [38]. Further work could investigate whether high anti-*L. donovani* IgG1 titre in asymptomatic patients is a potential biomarker for progression to active VL.

Seroepidemiological comparisons with more detailed and sophisticated technological analysis of patient profiles is clearly a promising approach to defining precise, robust and widely applicable biomarkers.

Based on analysis and interpretation of our results, in conjunction with review of the relevant literature, we conclude that:

1. Retention of high anti-*L. donovani* IgG1 titre (or presumably rising titre) 6 months after chemotherapy (but not immediately after chemotherapy) is an indicator of treatment failure and relapse (however this is not likely to hold true for immunocompromised HIV co-infected patients, in whom antibody responses may be impeded). Patients with such a serological profile require follow-up and alternative treatment or will almost certainly succumb to fatal VL.
2. Most importantly we have shown that high anti-*L. donovani* IgG1 levels 6 months after treatment, and in comparison with preceding pre- or shortly post-treatment levels, are amenable to detection with a simple lateral flow POC applicable RDT. Nevertheless, such RDTs should not be applied at the exclusion of concomitant clinical evaluation.

Further investigation of seroepidemiological indicators is clearly justified to find even better alternative biomarkers of clinical status, in particular to identify asymptomatic progressors to VL, as well as to distinguish cure from treatment failure and relapse.

## Supporting Information

Table S1 **Single (unpaired) samples used in ELISA IgG subclass comparisons and clinical status of the Indian and Sudanese patient groups.**
(DOCX)

Checklist S1 **STROBE Checklist.**
(DOCX)
