# Supplementary material for: IgG1 as a Potential Biomarker of Post-chemotherapeutic Relapse in Visceral Leishmaniasis, and Adaptation to a Rapid Diagnostic Test
Source: PLoS Negl Trop Dis. 2014 Oct 23;8(10):e3273. doi: 10.1371/journal.pntd.0003273 (PMC4207679; doi:10.1371/journal.pntd.0003273)
Supplement: Table S1 — Single (unpaired) samples used in ELISA IgG subclass comparisons and clinical status of the Indian and Sudanese patient groups. (DOCX) [file pntd.0003273.s001.docx]

**Table S1. Single (unpaired) samples used in ELISA IgG subclass comparisons and clinical status of the Indian and Sudanese patient groups.**

| Patient group | n | IgG1 | | IgG2 | | IgG3 | | IgG4 | |
| --- | --- | --- | --- | --- | --- | --- | --- | --- | --- |
| **Trial 1 India** |  | % positive | Mean A_490_ (95% CI) | % positive | Mean A_490_ (95% CI) | % positive | Mean A_490_ (95% CI) | % positive | Mean A_490_ (95% CI) |
| Active VL | 20 | 90.0 (18/20) | 1.47 (0.90 -2.03) | 5.0 (1/20) | 0.10 (0.08-0.12) | 75.0 (15/20) | 0.23 (0.16-0.31) | 0.0 (0/20) | 0.09 (0.07-0.10) |
| Cured VL | 21 | 33.3 (7/21) | 0.28 (0.15 – 0.40) | 0.0 (0/21) | 0.09 (0.08-0.10) | 14.3 (3/21) | 0.10 (0.06-0.14) | 4.8 (1/21) | 0.10 (0.7-0.13) |
| Relapsed VL | 19 | 84.2 (16/19) | 1.74 (1.17-2.31) | 15.8 (3/19) | 0.11 (0.09-0.14) | 52.6 (10/19) | 0.21 (0.14-0.29) | 5.3 (1/19) | 0.10 (0.08-0.12) |
| Other diseases | 20 | 0.0 (0/20) | 0.10 (0.08-0.11) | 0.0 (0/20) | 0.09 (0.08-0.11) | 5.0 (1/20) | 0.08 (0.07-0.09) | 5.0 (1/20) | 0.09 (0.06-0.13) |
| Endemic healthy control (seronegative) | 20 | 5.0 (1/20) | 0.09 (0.08-0.11) | 0.0 (0/20) | 0.10 (0.08-0.11) | 5.0 (1/20) | 0.08 (0.07-0.09) | 0.0 (0/20) | 0.10 (0.08-0.11) |
| **Trial 2 (Expanded) India** |  |  |  |  |  |  |  |  |  |
| Active VL | 46 | 67.4 (31/46) | 0.61 (0.47-0.75) | 4.3 (2/46) | 0.11 (0.10-0.12) | 28.3 (13/46) | 0.13 (0.12-0.15) | 40.0 (8/20) | 0.12 (0.11-0.14) |
| Cured VL | 28 | 3.6 (1/28) | 0.15 (0.10-0.20) | 7.1 (2/28) | 0.11 (0.09-0.12) | 3.6 (1/28) | 0.10 (0.08-0.11) | 35.7 (10/28) | 0.14 (0.11-0.16) |
| Relapsed VL | 35 | 71.4 (25/35) | 0.70 (0.49-0.92) | 0.0 (0/35) | 0.09 (0.08-0.10) | 25.7 (9/35) | 0.12 (0.10-0.15) | 2.9 (1/35) | 0.09 (0.08-0.10) |
| post kala-azar dermal leishmaniasis | 24 | 45.8 (11/24) | 0.49 (0.34-0.64) | 20.8 (5/24) | 0.15 (0.12-0.17) | 8.3 (2/24) | 0.12 (0.10-0.14) | 0.0 (0/24) | 0.10 (0.09-0.11) |
| Asymptomatic (seropositive) | 28 | 14.3 (4/28) | 0.24 (0.17-0.31) | 35.7 (10/28) | 0.15 (0.12-0.17) | 53.6 (15/28) | 0.16 (0.13-0.19) | 75.0 (21/28) | 0.18 (0.14-0.22) |
| Other diseases | 28 | 0.0 (0/28) | 0.12 (0.10-0.14) | 0.0 (0/28) | 0.11 (0.10-0.12) | 14.3 (4/28) | 0.10 (0.09-0.12) | 3.6 (1/28) | 0.09 (0.08-0.10) |
| Endemic healthy control (seronegative) | 32 | 3.1 (1/32) | 0.12 (0.09-0.15) | 3.1 (1/32) | 0.09 (0.08-0.10) | 3.1 (1/32) | 0.08 (0.07-0.09) | 3.1 (1/32) | 0.09 (0.08-0.09) |
| **Trial 1 Sudan** |  |  |  |  |  |  |  |  |  |
| Active VL | 47 | 57.4 (27/47) | 0.76 (0.60-0.92) | 6.4 (3/47) | 0.10 (0.08-0.11) | 48.9 (23/47) | 0.23 (0.19-0.28) | 6.4 (3/47) | 0.08 (0.08-0.09) |
| Treated VL^a^ | 22 | 4.6 (1/22) | 0.19 (0.13-0.25) | 0.0 (0/22) | 0.07 (0.06-0.08) | 4.6 (1/22) | 0.09 (0.07-0.11) | 0.0 (0/22) | 0.07 (0.06-0.07) |
| post kala-azar dermal leishmaniasis | 23 | 4.3 (1/23) | 0.18 (0.11-0.25) | 0.0 (0/23) | 0.07 (0.06-0.08) | 0.0 (0/23 | 0.09 (0.08-0.09) | 0.0 (0/23) | 0.07 (0.07-0.08) |
| DAT positive | 30 | 3.3 (1/30) | 0.15 (0.11-0.19) | 0.0 (0/30) | 0.09 (0.08-0.10) | 0.0 (0/30) | 0.09 (0.08-0.10) | 3.3 (1/30) | 0.08 (0.08-0.090 |
| Endemic healthy control (seronegative) | 12 | 0.0 (0/12) | 0.20 (0.13-0.26) | 0.0 (0/12) | 0.08 (0.07-0.10) | 0.0 (0/12) | 0.08 (0.06-0.11) | 0.0 (0/12) | 0.07 (0.06-0.08) |

^a^ treated, not in recent past, but time of treatment unknown
